# Adjunct Surgical Middle Meningeal Artery Obliteration for Recurrent Chronic Subdural Hematoma: Case Series and Technical Note

**DOI:** 10.1227/ons.0000000000001808

**Published:** 2025-10-10

**Authors:** Thomas Petutschnigg, Andreas Raabe, Philippe Schucht

**Affiliations:** Department of Neurosurgery, Inselspital Bern University Hospital, University of Bern, Bern, Switzerland

**Keywords:** Hematoma, Subdural, Chronic, Meningeal arteries, Burr-hole surgery, Middle meningeal artery obliteration, Case series, Technical note

## Abstract

**BACKGROUND AND OBJECTIVES:**

Recurrence after surgical treatment of chronic subdural hematoma (CSH) remains a clinical challenge. Endovascular occlusion of the middle meningeal artery (MMA) through embolization has emerged as an adjunctive procedure to reduce recurrence. We present a case series in which we performed navigated surgical obliteration of the MMA (soMMA) through a third burr hole as an alternative treatment.

**METHODS:**

In line with the Preferred Reporting of Case Series in Surgery guidelines for surgical case series, we retrospectively analyzed 4 patients with symptomatic recurrent CSH, who received for revision an additional navigated pterional burr hole for soMMA. We report the surgical anatomy, technical considerations, clinical outcomes and potential indications.

**RESULTS:**

All patients were male, multimorbid, with a mean age of 83 years, and presented with recurrent symptomatic unilateral or bilateral CSH. Revision with adjunct soMMA was performed between 10 and 90 days after index surgery. soMMA was safe, easily integrated into the workflow, and no further recurrences occurred. All patients regained full function and remained asymptomatic at last follow-up.

**CONCLUSION:**

Navigated adjunct soMMA through a pterional burr hole is a viable and cost-effective option for the management of recurrent CSH. This technique may be especially valuable for patients with contraindications for endovascular MMA embolization and for centers lacking endovascular resources.

**ABBREVIATIONS:**
CSH: chronic subdural hematoma
eMMAE: endovascular MMA embolization
LMWH: low-molecular-weight heparin
MMA: middle meningeal artery
RCT: randomized controlled trial
soMMA: surgical obliteration of the MMA.

Chronic subdural hematoma (CSH) is one of the most common neurosurgical conditions. Its rising incidence is attributed to aging populations, widespread use of antiplatelet/anticoagulant medication, and increased use of neuroimaging.^1-5^ Surgical evacuation with closed drainage systems remains the standard treatment of symptomatic CSH and is associated with low morbidity.^6-11^ However, symptomatic recurrence continues to affect a notable proportion of patients.^8,10,12^ Recurrence necessitates revision surgery, underscoring the need for strategies targeting the underlying pathophysiology.

A re-emerged view on the pathogenesis of CSH^13,14^ has prompted a paradigm shift in treatment.^15,16^ Rather than being viewed solely as a static collection of blood, CSH is recognized as a dynamic, inflammatory process centered on a neomembrane encapsulating the hematoma. This membrane harbors fragile, newly formed vessels that are highly susceptible to recurrent microhemorrhages, perpetuating a vicious cycle of hematoma expansion and recurrence.^17,18^ This revised pathophysiological model has enabled endovascular middle meningeal artery (MMA) embolization (eMMAE), which aims to arrest the inflammatory cycle by disrupting its pathological neovasculature.^19,20^ Recent randomized controlled trials (RCTs) have investigated the efficacy of eMMAE, marking a significant advancement in the management of CSH.^21-24^ Yet, despite its promise, eMMAE remains resource-intensive, costly, not without risk and its long-term efficacy is still being established.^25,26^

Surgical obliteration of the MMA (soMMA) may offer a practical alternative in this context.^27-29^ Adding MMA obliteration at the time of evacuation may simultaneously address mass effect and its underlying vascular driver. Despite this logical appeal,^27,28^ clinical evidence of soMMA is sparse, limited to a single previous case series,^29^ even though the anatomic considerations to reliably target the MMA were already meticulously defined over a century ago.^30-32^ In this case series, we describe a technical approach for navigated, adjunctive soMMA in patients with recurrent CSH and significant comorbidities.

## METHODS

We report a retrospective case series of 4 elderly patients with CSH who underwent revision surgery with adjunctive navigated soMMA at our neurosurgical department between 2023 and 2024. The study was in line with the Preferred Reporting of Case Series in Surgery guidelines for reporting surgical case series.^33^ In accordance with our internal protocol, all patients initially received standard treatment with 2 burr holes per affected side under general anesthesia, irrigation, and subgaleal drainage. No scheduled postoperative computed tomography (CT) or MRI follow-up was performed.^11^ Recurrence was defined by the reappearance of neurological symptoms, prompting imaging and subsequent intervention if indicated.

Clinical data were extracted from electronic medical records, including demographic characteristics, comorbidities, anticoagulation (antiplatelet/anticoagulation) therapy, neurological presentation, imaging findings, surgical details, postoperative course, and follow-up outcomes.

### Adjunct Pterional Burr Hole for soMMA

**FIGURE 1.**
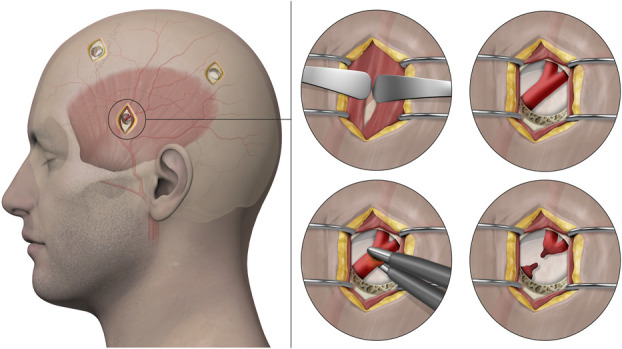
**Left**, Schematic lateral view of a patient's head depicting 2 standard convexity burr holes for hematoma evacuation alongside the pterional burr hole for targeted soMMA. **Right**, Here, the temporalis muscle is retracted using fish-hooks, and a standard trephination is performed. Beneath the pterional burr hole, the underlying MMA branch is coagulated using bipolar cautery. MMA, middle meningeal artery; soMMA, surgical obliteration of the MMA. *© 2025, Inselspital, Bern University Hospital, Department of Neurosurgery. All rights reserved*.

**FIGURE 2.**
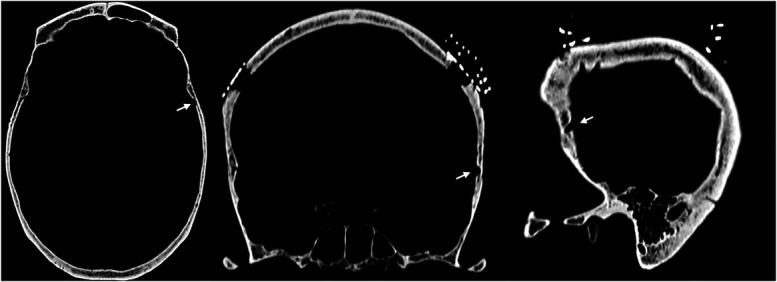
CT bone window of patient 4. The MMA sulcus (arrows) is clearly visible on all 3 planes. CT, computed tomography; MMA, middle meningeal artery.

For soMMA, preoperative CT imaging in the bone window was used for navigation, with no additional sequences required. Navigation was performed using the Brainlab Cranial Navigation software (Brainlab AG) within the standard cranial planning module. In 2 cases, the SmartBrush tool (version 4.5) was used to facilitate surgical planning by marking the MMA sulcus/canal at its temporopterional segment, which was clearly visible on the CT bone window,^34,35^ targeting the anterior division of the MMA (Figures 1 and 2). This target point was selected based on previous anatomic considerations, which identified it as an optimal trephination site for soMMA.^27,28,30-32,36^

Anticoagulation therapy was managed individually based on thromboembolic risk, indication (mechanical valve, prior stroke, coronary artery disease), and multidisciplinary input. Reversal agents were used in patients with elevated international normalized ratio, and bridging with low-molecular-weight heparin (LMWH) was implemented postoperatively when appropriate.

Patients were monitored clinically during hospitalization and at follow-up intervals of 1 month, 3 months, and up to 11 months after the index surgery. Functional outcomes were assessed based on neurological examination, symptom resolution, and gait function.

### Ethics

This retrospective analysis was conducted in accordance with the Swiss Human Research Act. All patients gave general consent, and ethics committee approval was waived.

## RESULTS

### Patient 1

An 84-year-old patient with a medical history of arterial hypertension and dyslipidemia, on antiplatelet therapy (aspirin) because of a previous transient ischemic attack, presented to the emergency department. The Glasgow Coma Scale (GCS) score was 14, notable for disorientation and right-sided hemiparesis (Medical Research Council 3). Cranial MRI revealed bilateral CSH.

The patient underwent bilateral 2 burr-hole trephination with placement of subgaleal drains. Clinical recovery was uneventful, and the patient was discharged to a geriatric rehabilitation facility, neurologically intact.

One month later, the patient reported new-onset gait disturbance and cognitive decline. CT imaging demonstrated recurrent bilateral CSH. A revision procedure was performed, consisting of reopening the existing burr holes bilaterally and placement of subgaleal drains. The patient was discharged home on postoperative day 5 with complete resolution of symptoms.

**FIGURE 3.**
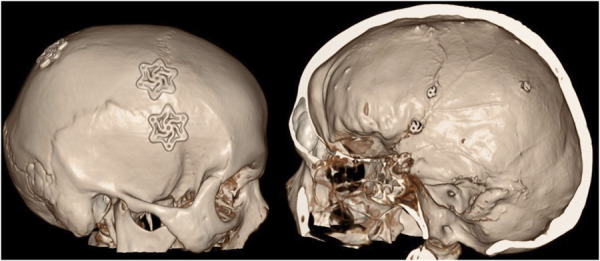
**Left**, A right-lateral 3D CT reconstruction of patient 1 demonstrating the position of the navigated pterional burr hole, here covered with a titanium plate. **Right**, A view from the inside-out perspective, displaying the inner skull surface, confirming that the burr hole was precisely positioned at the pterional MMA sulcus. Note the impressions of the MMA branches on the inner skull surface and how the standard frontal burr hole already targeted a distal branch. 3D, 3-dimensional; CT, computed tomography; MMA, middle meningeal artery.

Three months following the index surgery the patient presented with a mild motor drift of the left upper limb. The CT scan showed progression of the right-sided hematoma. A third surgical intervention was performed, targeting the right side: reopening of the burr holes with placement of a subgaleal drain and adjunctive soMMA through a navigated pterional burr hole (Figure 3). The patient was discharged on postoperative day 4 after full functional recovery. Aspirin was reintroduced, and follow-up for 8 months postoperatively found no recurrence or neurological deficits.

### Patient 2

An 85-year-old patient presented to the emergency department with a GCS score of 14 and impaired gait. The patient had a history of recurrent vertebrobasilar ischemic strokes for which he was on clopidogrel therapy, as well as chronic obstructive pulmonary disease (Global Initiative for Chronic Obstructive Lung Disease, stage 2), chronic kidney disease, and arterial hypertension neurological examination revealed generalized weakness without lateralization. Cranial CT imaging demonstrated a left-sided CSH.

The patient underwent left-sided 2 burr-hole trephination with placement of a subgaleal drain. Postoperative recovery was uneventful, and the patient was discharged home on postoperative day 4 with complete resolution of symptoms.

Two weeks later, the patient returned to the emergency department with new-onset slurred speech. The GCS is again at 14, with no additional focal neurological deficits. CT imaging revealed a residual left-sided CSH. Revision surgery was performed through reopening of the previous burr holes with placement of a subgaleal drain. The patient was discharged home after 6 days without any residual neurological deficits.

Two weeks later, the patient presented again, with worsening gait instability and aphasia. Repeat CT revealed persistent residual hematoma on the left side. A third revision was undertaken, including reopening of the previous burr holes, subgaleal drainage, and adjunctive navigated soMMA. Clopidogrel therapy was resumed. Clinical and radiological follow-up continued for 11 months after the index procedure, with no evidence of recurrence or neurological deficits.

### Patient 3

A 79-year-old patient was referred to the emergency department for evaluation of headache, gait disturbance, bilateral lower limb weakness, and aphasia. The patient had a complex medical history including cardiac arrhythmia, congenital epilepsy, mitral valve insufficiency, and artificial mechanical aortic valve, and was on long-term anticoagulation with phenprocoumon (International Normalized Ratio, 2.4 on admission). Cranial CT imaging revealed bilateral CSH with mass effect.

Anticoagulation was reversed preoperatively using prothrombin complex concentrate and vitamin K. The patient subsequently underwent bilateral burr-hole trephination with subgaleal drainage. Postoperative recovery was favorable, with complete resolution of neurological deficits. On postoperative day 3, the patient was transferred to the referring hospital, neurologically intact and without aphasia or motor deficits. Owing to the mechanical heart valve, subtherapeutic LMWH was initiated in the early postoperative period.

Ten days after the initial surgery, the patient represented symptomatic, with a recurrent left-sided hematoma causing mass effect. Revision surgery was performed, including reopening of the previous burr holes, placement of a subgaleal drain, and navigated soMMA. LMWH was later increased to therapeutic levels and subsequently bridged back to phenprocoumon. Follow-up for 3 months after the index procedure found no recurrence or neurological symptoms.

### Patient 4

An 83-year-old patient presented with preserved consciousness (GCS 15), progressive headache, gait and balance disturbance. The patient had a history of myocardial infarction treated with coronary stenting and ongoing clopidogrel therapy, extracranial carotid artery stenosis, previous transient ischemic attack, and a recent mild traumatic brain injury (1 month earlier). Cranial MRI revealed bilateral CSH.

The patient underwent bilateral burr-hole trephination with placement of subgaleal drains. Recovery was uneventful, and the patient was discharged to a geriatric rehabilitation facility neurologically intact.

Twenty days postoperatively, the patient developed renewed gait disturbances and ataxia. CT imaging demonstrated recurrence of the left-sided CSH. A revision procedure was performed with re-evacuation of the hematoma and placement of a subgaleal drain. The patient was discharged again to rehabilitation after 3 days, with resolution of symptoms and improved gait.

**FIGURE 4.**
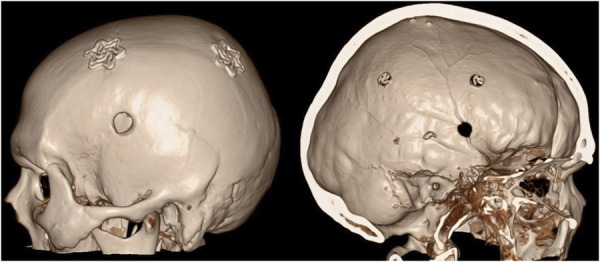
**Left**, A left-lateral 3D CT reconstruction of patient 4 demonstrating the position of the navigated pterional burr hole slightly posterior compared to Figure 3, here without a cover plate. **Right**, A view from the inside-out perspective, displaying the inner skull surface, demonstrating projection of the pterional burr hole on the MMA course. 3D, 3-dimensional; CT, computed tomography; MMA, middle meningeal artery.

One month later, clopidogrel therapy was resumed. However, 3 months after the index procedure, the patient presented again with vertigo and worsening gait disturbance. CT confirmed a recurrent left-sided CSH. Revision surgery was performed through reopening of the existing burr holes, subgaleal drainage, and navigated soMMA through a pterional burr hole (Figure 4).

The patient was discharged home on postoperative day 8 with improved gait and no residual ataxia. Follow-up 5 months after the initial surgery confirmed a stable condition without neurological deficits. All cases are summarized in Table.

**TABLE.** Summary of Cases

Patient	Sex	Age (y)	Comorbidities	Anticoagulation (antiplatelet/anticoagulation)	Initial surgery	Recurrence	Revision surgery	Outcome	Follow-up duration (mo)
1	`M	84	TIA, HTN, dyslipidemia	Aspirin	Bilateral burr holes + subgaleal drain	Twice (1 and 3 mo)	Navigated soMMA (third surgery)	Full recovery	8
2	M	85	Vertebrobasilar stroke, CKD, COPD, HTN	Clopidogrel	Left burr holes + subgaleal drain	Twice (2 and 4 wk)	Navigated soMMA (third surgery)	Full recovery	11
3	M	79	Mechanical valve, arrhythmia, epilepsy, MI	Phenprocoumon	Bilateral burr holes + subgaleal drain	Day 10	Navigated soMMA (second surgery)	Full recovery	3
4	M	83	MI, carotid stenosis, TIA	Clopidogrel	Bilateral burr holes + subgaleal drain	Twice (day 20 and 3 mo)	Navigated soMMA (third surgery)	Full recovery	5

CKD, chronic kidney disease; COPD, chronic obstructive pulmonary disease; HTN, arterial hypertension; M, male; MMA, middle meningeal artery; MI, mitral valve insufficiency; soMMA, surgical obliteration of the MMA; TIA, transient ischemic attack.

## DISCUSSION

Managing CSH in elderly, especially frail patients on anticoagulation remains a significant challenge because of high recurrence rates and complexity of treatment decisions.^37^ Despite a wealth of literature on CSH and the growing enthusiasm for eMMAE, soMMA has received remarkably little attention. Notably, surgical approaches targeting the MMA were already being rigorously studied as early as the 1890s.^30,36^ However, only recently a handful of publications have considered this approach for CSH^27,28^ and to our knowledge, aside from Cardoso et al,^29^ our series is the only one to describe its practical application. This is in part because the MMA as a therapeutic target is a relatively recent concept, whose momentum has been driven by interventionalists and industry interest in endovascular innovation. Nevertheless, this underlines the novelty and the potential relevance of soMMA, particularly in selected clinical scenarios where endovascular treatment is not feasible or desirable.

### Surgical Anatomy

The MMA originates from the internal maxillary artery and enters the cranial cavity through the foramen spinosum. It courses anterolaterally along the inner surface of the temporal bone, passing across the inner pterion within a sulcus or bony canal, before bifurcating into an anterior and posterior division.^28,36,38,39^ The anterior division is typically larger, acts as main branch, and ascends along the parietal bone toward the bregma, parallel the central sulcus. The smaller posterior branch extends toward parieto-temporo-occipital regions.^30,34,35^ As early as the 19th century, surgeons such as Krönlein, Steiner, Kocher, Vogt, and Witherle conducted detailed craniometric studies to establish reliable trephination landmarks for targeting the MMA, mainly for the treatment of epidural hematomas and Gasserian ganglion procedures. They all found that the anterior branch of the MMA can be reliably targeted at the pterion.^30-32^ Now more recent studies regained this idea in the context of CSH.^27,28^ Although foundational craniometric research has been essential, the MMA sulcus, as prominent surrogate landmark for the pterion, can also be visualized intraoperatively using fluoroscopy.^29^ However, navigation-assisted targeting with conventional CT imaging provides even more reliable localization of the MMA sulcus/canal (Figure 2).

Yet, some uncertainty remains regarding the proximity to the facial nerve.^27,28^ Synthesizing multiple existing anatomic studies across the neurosurgical and craniofacial literature demonstrates that the pterion reliably lies posterior and superior to the facial nerve's frontal branch.^38-42^

### Technical Aspects

Unlike endovascular approaches that enable proximal MMA embolization, soMMA acts more distally at the MMA. However, recent evidence suggests no difference in hematoma resolution between proximal and distal eMMAE,^43^ indicating that occlusion anywhere along the MMA can disrupt the vicious cycle. Although distal penetration and casting of the whole MMA is often emphasized in the eMMAE literature,^20,44^ the need for this remains theoretical, with no proven therapeutic benefit. Nevertheless, this could reflect a limitation of soMMA, as it may induce a different physiological response. Yet, soMMA offers the advantage of addressing both hematoma evacuation and MMA occlusion in a single procedure, eliminating the need for additional anesthesia, an AngioSuite, or hybrid operating room.

Our approach differs from that of Cardoso et al^29^ in several respects. They used mainly 1 single pterional burr hole for index CSH surgery, we drilled an adjunctive third burr hole exclusively at revision surgery. Notably, Cardoso et al^29^ subsequently drained the hematoma through this pterional burr hole, while we only used the adjunctive burr hole for hematoma evacuation in 1 case. In all other cases, the preexisting convexity burr holes were used for hematoma evacuation and irrigation. We believe that this strategy maximizes the procedural safety, avoids damage of the brain parenchyma and the risk of postoperative pneumocephalus.

Despite technical differences, neither Cardoso et al^29^ nor we observed any facial nerve injury related to the targeted pterional skin incisions. To further mitigate risks, the skin incisions could be aligned parallel to the nerve's typical posterior-inferior to anterior-superior course. Cosmetic outcomes were satisfactory, with well-healed, discreet incisions concealed within the hairline.

### Indication and Future Directions

eMMAE has gained further robust support from recent randomized trials and meta-analyses.^21-23^ Moreover, European and North-American multidisciplinary consensus statements already recommend eMMAE for patients with recurrent CSH.^15,16^ However, eMMAE exposes patients to a new set of procedure-related risks including access site complications, catheter-associated complications, and nontargeted embolizations.^24,26,45-47^ All of these are compounded by age-related factors such as atherosclerosis, tortuous arterial anatomy and vascular fragility^26^ and anatomic variations.^20,47^ In fact, in a RCT, approximately 12% of patients allocated to eMMAE were ultimately not treated because of dangerous anastomoses, catheterization failure, or stroke during catheter navigation.^24^ By contrast, soMMA could theoretically lead to iatrogenic facial nerve injury, epidural hematoma, parenchymal damage, and temporal muscle injury. Although no procedure-related complications were observed in the current small series,^29^ they should still be considered.

From an economic perspective, eMMAE is significantly more costly than standard surgical evacuation, due to the use of expensive embolic agents and specialized endovascular equipment.^25^ These economic considerations are not confined to low-resource settings, since high-income healthcare systems are increasingly strained by rising volumes of medical procedures and aging population.^16^ Notably, 2 of the 4 recent RCTs were industry sponsored, underscoring the commercial interests driving the widespread adoption of eMMAE.^21-24^ By contrast, soMMA uses existing surgical infrastructure and requires only a trephine and basic (micro-)surgical instruments, making it a more economically sustainable alternative, but 1 that remains underexplored. However, soMMA could already be considered as an option for treatment of recurrent CSH in patients with contraindications to eMMAE.

The optimal timing of adjunctive treatments, whether at the index procedure or reserved for recurrence, remains a matter of debate.^15,19,47,48^ In our series, soMMA was performed exclusively in patients with recurrence considered at high risk of further relapse, whereas Cardoso et al^29^ reported its application already at the index surgery. Similarly, for eMMAE, future studies and subgroup analysis may need to find robust patient selection criteria for adjunctive therapy^15,19,47,48^ to avoid overtreatment.

### Limitations

As this is a descriptive case series, the applicability of our results is limited. The reproducibility and broader efficacy of soMMA require validation in larger series and trial. Therefore, while our work contributes to the evolving dialog on CSH management, further research is essential to define its role as an alternative to endovascular interventions.

## CONCLUSION

In this small case series, adjunctive navigated soMMA through a pterional burr hole seemed safe and feasible for revision surgery for recurrent CSH. The approach offers potential practical advantages over endovascular embolization, including lower cost and direct intraoperative application without the need for a hybrid operating room or AngioSuite. Although these initial observations are promising, further studies are needed to validate its efficacy and broader applicability.

## COMMENTS

The authors present a fascinating case series of multimorbid patients who present with recurrent chronic subdural hematoma after initial burr-hole evacuation. After multiple recurrences, hematoma resolution in these patients was achieved with surgical occlusion of the middle meningeal artery, utilizing stereotactic localization and microsurgical technique. This case series is timely given the mounting evidence supporting endovascular MMA embolization as primary or secondary treatment for cSDH. Questions remain concerning comparative procedural efficacy (ie, against standard MMA embolization), when this procedure should be applied (ie, at initial procedure or with recurrence), and overall comparative procedural risk. Two concepts are clear: (1) This approach has the potential to be more cost effective than an additional endovascular procedure, and (2) 1 or multiple prospective trials are necessary to elucidate the role of this procedure in chronic subdural hematoma management. The authors should be commended for proposing a potentially efficacious, cost-reducing solution to a complex problem.

**Randy S. Bell**

*Bethesda, Maryland, USA*
